# An Unusual Complication after Shoulder Hemiarthroplasty

**DOI:** 10.1155/2013/759193

**Published:** 2013-04-04

**Authors:** Oliver D. Stone, S. J. Breusch

**Affiliations:** Edinburgh Royal Infirmary, Orthopaedic Department, 51 Little France Crescent, Old Dalkeith Road, Edinburgh, Midlothian EH16 4SA, UK

## Abstract

Hemiarthroplasty of the shoulder can be a safe and an effective treatment for pain in patients with rheumatoid arthritis. Many complications have been previously described in the literature; the most common of which are dislocation, loosening, periprosthetic fractures, and infection. We report a patient who presented with a discharging sinus over the tip of the acromium which was created by the displacement of the prosthesis and erosion of the AC joint and distal clavicle. The erosion of the distal clavicle and AC joint caused the remaining proximal clavicle to become mobile and displaced posteriorly; this spike of clavicle was then able to penetrate the trapezius muscle and eventually the skin causing an aseptic sinus. This was successfully treated with the exploration and excision of the distal 2 cm of the clavicle.

## 1. Introduction

Shoulder hemiarthroplasty can be an effective way of treating arthritis, and, in the UK, approximately 1500 are performed each year [1]. Shoulder hemiarthroplasty is primarily performed as a pain relieving procedure in arthritis with improvements in function usually noted [2] or as a primary treatment for certain severe grades of proximal humerus fractures.

## 2. Case Report

The patient in question was a 90-year-old female who suffered with a destructive form of erosive rheumatoid arthritis. At the time of her presentation she was living alone in a 3-bedroom flat with a substantial package of care and had become in recent months almost wheelchair bound.

At the age of 76, she had a left Neer shoulder hemiarthroplasty for avascular necrosis of her humeral head following a dislocation. For 10 years she had satisfactory level of function and was pain-free; however, at the age of 86, she was experiencing pain in her shoulder as was seen in the out-patient clinic. Clinically, she had severe rotator cuff dysfunction with anterior and superior displacement of the prosthesis, but no intervention was recommended at this time.

The patient then represented at the age of 89 with a 6-week history of her left shoulder becoming increasingly painful and stiff and with a small discharging wound over the posterior aspect of her left shoulder.

On presentation, the patient was apyrexial with normal observations and felt systemically well. There was a 1 cm sinus over the posterior aspect of her shoulder in the supra-spinatus fossa just medial to the acromium process. The wound was discharging moderate amounts of serous fluid which was causing the patient some distress and also skin irritation.

**Figure 1:**
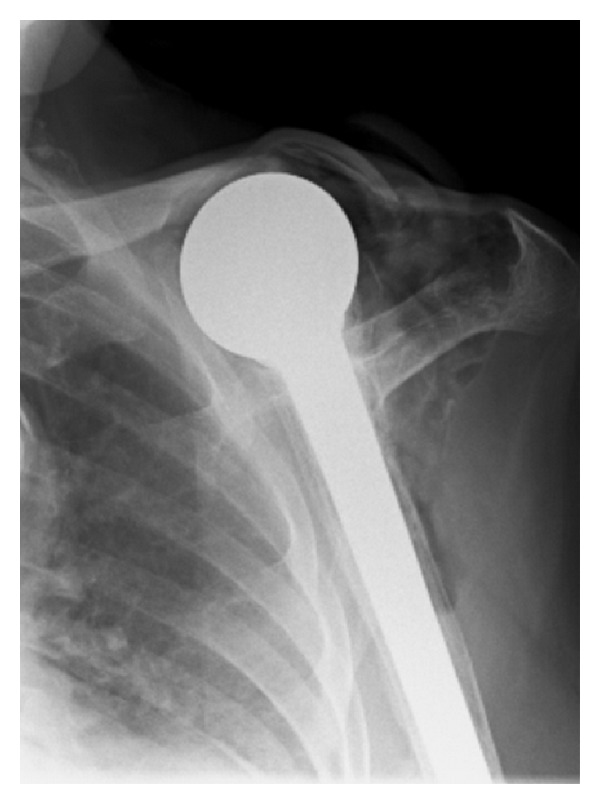
AP left shoulder on initial presentation February 12, 2009. Note the distal clavicle destruction, gas in the soft tissues, and the abnormal position of the prosthesis.

She was seen by her GP who organised some investigations including inflammatory markers and X-rays (see Figure 1). When compared to previous X-rays, a grossly abnormal left shoulder was seen with the dislocation of the humeral head. There was a loss of the lateral 3rd of the left clavicle with bony destruction of the clavicle and glenoid. There was overlying soft tissue opacification containing gas, and the overall appearances were suggestive of aggressive osteomyelitis.

Her blood tests that were taken at the time were normal, WCC 6.2, ESR 25, and an CRP of 2. Swabs and cultures were taken at this initial presentation and came back with no growth. 

She was then taken to theatre for exploration of the sinus, and large seroma was found; a bony prominence was seen and was identified as what remained of the clavicle which had been the cause of the perforation to the skin; this was trimmed back to prevent recurrence, and good soft tissue coverage was achieved. Tissue and fluid samples taken intraoperatively were cultured and later demonstrated no growth.

## 3. Discussion

The combination of the patient's hemiarthroplasty, rotator cuff pathology, and erosive rheumatoid arthritis led to her prosthesis being displaced in an anterior and superior direction; the erosion of her distal clavicle by the mechanical action of her hemiarthroplasty led to the shortened remnant of clavicle being displaced posterior which then penetrated the skin and caused an aseptic sinus.

Throughout her presentation, she showed no signs of sepsis, her inflammatory markers were always normal, and all of the cultures were negative for organisms.

**Figure 2:**
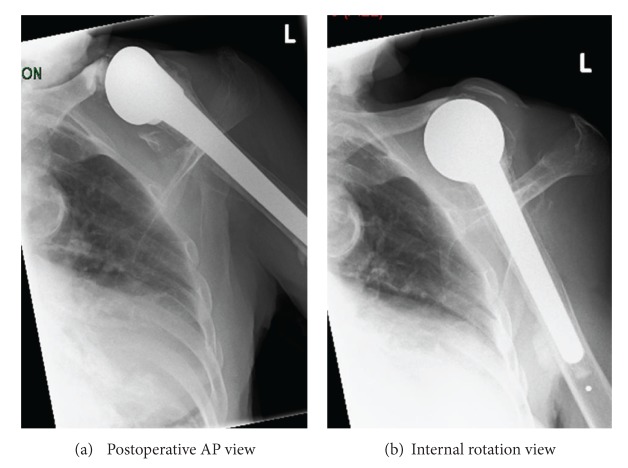
Postoperative AP and internal rotation views of the left shoulder. Note the trimmed clavicle and the lack of gas in the soft tissues following successful closure.

Once the distal clavicle was excised and the wound closed, it healed without complication, and the pain and discharge resolved. (See Figure 2 for postoperative radiograph).

This case demonstrates an unusual complication of a shoulder hemiarthroplasty in a rheumatoid patient which, to our knowledge, has not been described in the literature to date.
